# Development of a Stability-Indicating RP-UPLC Method for Rapid Determination of Metaxalone and its Degradation Products in Solid Oral Dosage Form

**DOI:** 10.3797/scipharm.1112-08

**Published:** 2012-02-21

**Authors:** Rakshit Kanubhai Trivedi, Mukesh C. Patel

**Affiliations:** 1 Analytical Research and Development, Integrated Product Development, Dr. Reddy’s Laboratories Ltd., Bachupally, Hyderabad-500 072, India; 2 P.S. Science and H.D. Patel Arts College, S.V. Campus, Kadi-382 715, Gujarat, India

**Keywords:** Skeletal muscle relaxant, Method validation, Forced degradation, Assay, Related substances, UV spectra, Chromatography

## Abstract

A simple, sensitive and reproducible reversed phase ultra performance liquid chromatography (RP-UPLC) coupled with a photodiode array detector method was developed for the quantitative determination of metaxalone (META) in pharmaceutical dosage forms. The method is applicable to the quantification of related substances and assay of drug product. Chromatographic separation was achieved on an Acquity^®^ HSS-T3 (100 mm x 2.1 mm, 1.7 μm) column. The optimized isocratic mobile phase consists of a mixture of water, methanol, acetonitrile and triethylamine in the ratio of 50:25:25:0.1 % v/v (pH adjusted to 6.3 with orthophosphoric acid). The eluted compounds were monitored at 230 nm for META assay and 205 nm for related substances, the flow rate was 0.3 mL/min, and the column oven temperature was maintained at 45°C. The developed method separated META from its two known and two unknown impurities within 6.0 min. Metaxalone was subjected to the stress conditions of oxidative, acid, base, hydrolytic, thermal and photolytic degradation. Metaxalone was found to degrade significantly in base stress condition, degrade slightly in oxidative stress condition and remain stable in acid, hydrolytic, thermal and photolytic degradation conditions. All impurities were well resolved from each other and from the main peak, showing the stability-indicating power of the method. The developed method was validated as per International Conference on Harmonization (ICH) guidelines.

## Introduction

Metaxalone, chemically 5-[(3,5-dimethylphenoxy)methyl]-1,3-oxazolidin-2-one, was first synthesized by Lunsford, *et al.* [1]. The chemistry of the metabolic products was proposed by Bruce *et al*. [2]. Metaxalone (marketed by King Pharmaceutical under the brand name SKELAXIN^®^, approved by the US FDA in 1962 [3]) is a muscle relaxant and useful to relieve the pain caused by strains, sprains and other musculoskeletal conditions. Its exact mechanism of action is not known, but it may be due to general central nervous system depression. It is considered to be a moderately strong muscle relaxant, with relatively low incidence of side effects, low toxicity [4] and no reports of major safety issues [5]. In addition, because of new indications it seems that this drug product will continue to attract attention [6–8]. Metaxalone exhibits increased bioavailability when taken with food [9].

UPLC is a new category of separation technique based upon well-established principles of liquid chromatography, which utilizes sub-2 μm particles for the stationary phase. These particles operate at elevated mobile phase linear velocities to affect dramatic increases in resolution, sensitivity and speed of analysis. Owing to its speed and sensitivity, this technique has gained considerable attention in recent years for pharmaceuticals and biomedical analysis. In the present work, this technology has been applied to the method development and method validation study of related substances and the assay determination of Metaxalone in pharmaceutical dosage forms.

Very few methods have appeared in the literature for the assay determination of META in bulk and pharmaceutical dosage forms by high-performance liquid chromatography (HPLC) [10–12]. Narasimha et *al.* [13], described method validation of META by using UV spectroscopy. Nirogi RV et *al.* [14], described a method for quantification of META in human plasma by HPLC coupled with tandem mass spectroscopy. META is not official as drug substance as well as drug product in the European Pharmacopoeia (Ph. Eur.) and United States Pharmacopoeia (USP). To the best of our knowledge, none of the currently available analytical methods can separate and quantify all the known related compounds and degradation impurities of META API and dosage forms. Furthermore, there is no stability-indicating HPLC/UPLC method reported in the literature that can adequately separate and accurately quantify META API and dosage forms. It is, therefore, felt necessary to develop a new rapid, stability-indicating method for the related substance determination and quantitative determination of META. We intend to opt for the faster technique UPLC for the said study. An attempt was made to determine whether UPLC can reduce analysis times without compromising the resolution and sensitivity.

**Fig. 1.**
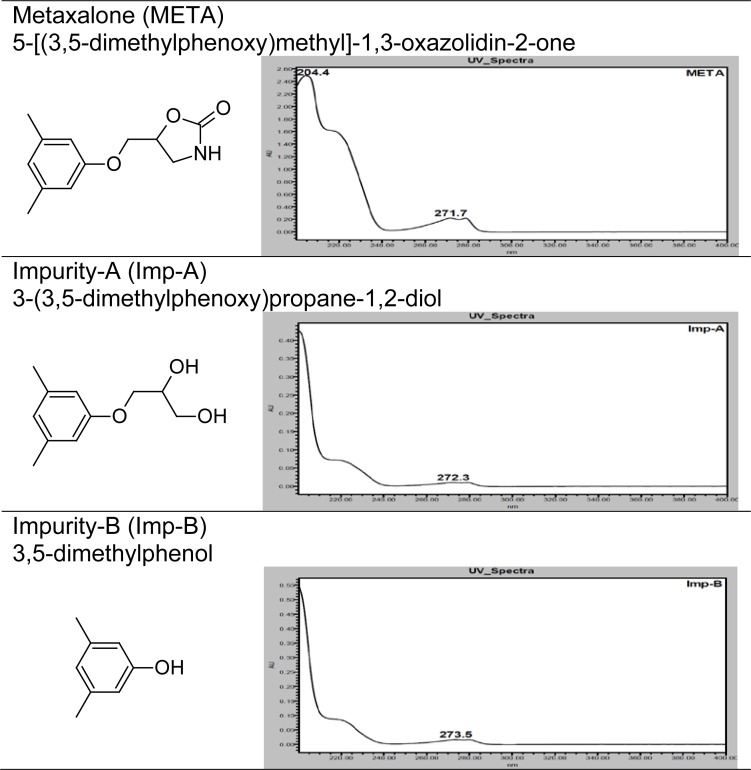
Chemical structure, UV spectrum and IUPAC name of META, Imp-A and Imp-B.

Therefore, a reproducible stability-indicating RP-UPLC method was developed for the quantitative determination of META and its two known impurities (Imp-A and Imp-B), this developed proposed method is also able to separate two unknown degradation products from interested compounds within 6.0 min. This method was successfully validated according to the ICH guidelines (Validation of Analytical Procedures: Test and Methodology Q2) [15]. Chemical structures, UV spectrums and IUPAC name of META, Imp-A and Imp-B are presented in Figure 1.

## Results and Discussion

### Method development and optimization

The main objective of the RP-UPLC method development was the finding of a rapid assay and related substances determination of Metaxalone in pharmaceutical formulation were: the method should be able to determine assay (AS) and related substances (RS) in single run and should be accurate, reproducible, robust, stability-indicating, filter compatible, linear, free of interference from blank / placebo / impurities / degradation products and straightforward enough for routine use in quality control laboratory.

The spiked solution of META (1000 μg/mL), IMP-A (1 μg/mL) and IMP-B (1 μg/mL) was subjected to separation by RP-UPLC. Initially the separation of all compounds was studied using water as a mobile phase-A (MP-A) and acetonitrile (ACN) as a mobile phase-B (MP-B) on a UPLC column (Eclipse Plus C18, RRHD, 50 × 2.1mm; 1.8μm) using a Waters (UPLC) system with the linear gradient program. The flow rate of 0.3 mL/min was selected with regards to the backpressure and analysis time as well. Various types of MP-A and MP-B were studied to optimize the method, which were summarized in Table 1 with the associated observations.

**Fig. 2.**
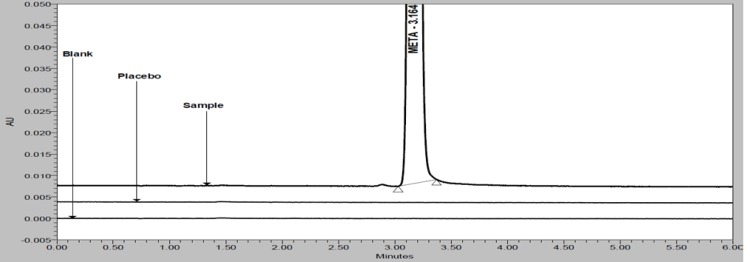
Expanded overlay chromatograms of blank, placebo and sample (at 230nm)

**Fig. 3.**
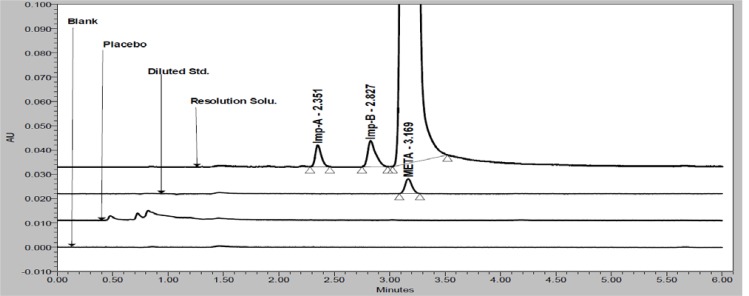
Overlay chromatograms of blank, placebo, diluted standard and spiked impurities along with analyte (at 205nm)

Based on above mobile phase selection experimental study, the optimized UPLC parameters were: flow rate 0.3 mL/min; column oven temperature 45°C; mixture of water, ACN, MeOH and TEA in the ratio of 50:25:25:0.1 v/v/v/v, respectively (pH adjusted to 6.3 with OPA). In order to achieve symmetrical peak shape of all substances and more resolution between META and Imp-B, different stationary phases were explored. Peak merging (Imp-B and META) and broad peak shape of META was observed with an Acquity BEH C8 (100 × 2.1 mm, 1.7μm) column. Poor resolution (Imp-B and META) and broad peak shape of META was observed with an Acquity BEH C18 (100 × 2.1 mm, 1.7μm) column. Finally, the desired separation with symmetrical peaks was obtained using Acquity HSS-T3 (100 × 2.1mm, 1.8μm) column. Column oven temperature was also studied and it was found that 45°C is more appropriate with respect to separation and peak shape. Based on compounds UV response, 230nm (for assay) and 205nm (for related substances) was found more appropriate for determination of META and its impurities from single run. META, Imp-A and Imp-B are well resolved from each other and there was no chromatographic interference observed due to blank and placebo in a reasonable time of 6.0 minutes (Figure 2 and 3).

### Analytical parameters and validation

After satisfactory development of the method it was subjected to method validation as per ICH guidelines [15]. The method was validated to demonstrate that it is suitable for its intended purpose by the standard procedure to evaluate adequate validation characteristics (system suitability, accuracy, precision, linearity, robustness, solution stability, filter compatibility and stability-indicating capability).

### Specificity

Specificity is the ability of the method to measure the analyte response in the presence of its potential impurities. Forced degradation studies were performed to demonstrate selectivity and stability-indicating capability of the proposed RP-UPLC method. Figures 2 and 3 show that there are not any interferences at the RT (retention time) of META due to blank, placebo and impurities. Stress studies were performed at concentration of 1000 μg/mL of META to provide the stability-indicating property and specificity of the proposed method.

**Fig. 4.**
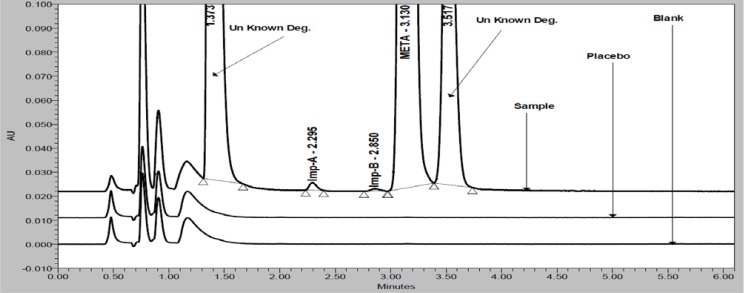
Overlay chromatograms (blank, placebo and drug product) of alkali degradation study.

**Fig. 5.**
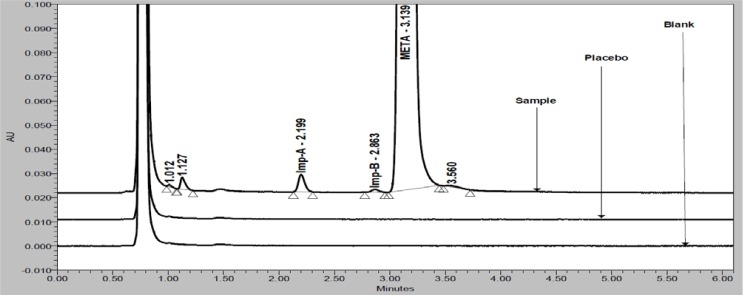
Overlay chromatograms (blank, placebo and drug product) of peroxide degradation study.

Forced degradation studies were performed by the stress conditions, UV light (254 nm for 10 days), heat (for 6h at 105 °C), acid hydrolysis (1 N HCl at 60 °C for 1h), water hydrolysis (at 60 °C for 2h), base hydrolysis (1 N NaOH at 60 °C for 30 min, Figure 4) and oxidation (30% H_2_O_2_ at 60 °C for 1h, Figure 5) to evaluate the ability of the proposed method to separate META from its degradation products. Degradation was not observed when META was subjected to acid, heat, photolytic and hydrolytic conditions. Significant degradation was observed when the drug product was subjected to base hydrolysis, and slight degradation was observed when the drug product was subjected to oxidative hydrolysis. Overlaid chromatograms (blank, placebo and drug product) of base hydrolysis and peroxide degradation study are presented in Figures 4 and 5. The purity of the peaks obtained from the stressed sample was verified using the PDA detector. The obtained purity angle was less than purity threshold for all the stressed samples. An assay of samples was performed by comparison with reference standards, and the mass balance [% assay + % known impurities + area % unknown impurities, at 205nm] for each of the stressed samples was calculated. The results from forced degradation study are given in Table 2.

### Precision

The system precision of the related substance (at 205 nm) method was verified by injecting six replicate injections of a standard solution containing META (1 μg/mL) and its two impurities of 1 μg/mL of each. The RSD (%) of the peak areas was calculated for each compound (system precision). Method precision experiments were conducted in six individual preparations of META (1000 μg/mL) spiked with 1 μg/mL each of the impurities and the RSD (%) for area percentage of each impurities was calculated. Precision of assay (at 230 nm) method was evaluated by performing six (n=6) independent assays of META tablet at 1000 mg/mL level against a qualified working standard. The RSD (%) of the six results was calculated. The intermediate precision of the assay and RS method was evaluated by different analyst, instrument and day. The RSD (%) of peak area of META, Imp-A and Imp-B in system precision was within 1.0% (Table 3). The RSD (%) results of META and its impurities for precision and intermediate precision are presented in (Table 4). These results confirmed the high precision of the method. As seen from this data, the acceptable system suitability parameters would be: resolution between Imp-B and META is not less than 1.5, theoretical plates are not less than 7000, tailing factor for META is not more than 2.0.

### Accuracy

The accuracy of an analytical procedure expresses the closeness of agreement between the true value and the observed value. The accuracy of the assay method for META was evaluated in triplicate (n=3) at the three concentrations of 500, 1000 and 1500 μg/mL (50, 100 and 150%) of drug product, and the recovery was calculated for each added (externally spiked) concentration. For all impurities, the recovery was determined in triplicate (n=3) for 0.5, 1.0 and 1.5 μg/mL (50, 100 and 150%) of the analyte concentration (1000 μg/mL) of the drug product, and the recovery of the impurities was calculated. The amount recovered was within ± 1.5 % (for assay) and ± 5.0 % (for related substances) of amount added, which indicates that there is no interference due to excipients present in pharmaceutical dosage forms. The results confirmed that the method is highly accurate (Table 5).

### Linearity of response

The linearity of an analytical method is its ability to elicit test results that are directly proportional, or by a well-defined mathematical transformation to the concentration of analyte in a sample within a given range. The detector response linearity for Imp-A, Imp-B and META were assessed by injecting nine separately prepared solutions covering the range of LOQ (0.1 μg/mL) to 2.0 μg/mL (LOQ, 0.2, 0.4, 0.6, 0.8, 1.0, 1.2, 1.5 and 2.0 μg/mL) of the normal analyte concentration (1 μg/mL). For META assay the response function was determined by preparing standard solutions at seven different concentration levels ranging from 500 to 1500 μg/mL (500, 700, 800, 1000, 1200, 1400 and 1500 μg/mL). The correlation coefficients, slopes and *y*-intercepts of the calibration curve were determined (Table 6). The correlation coefficient obtained was greater than 0.999 in both cases (Table 6).

### Limit of detection (LOD) and limit of quantification (LOQ)

The LOD and LOQ for META and its impurities were determined at a signal to noise ratio of 3:1 and 10:1, respectively, by injecting a series of dilute solutions with known concentrations. Precision study was also carried out at the LOQ level by injecting six (n=6) individual preparation and calculating the % RSD of the area for each impurity and for META. The determined limit of detection, limit of quantification and precision at LOQ levels for META, Imp-A and Imp-B are presented in Table 7.

### Robustness

**Fig. 6.**
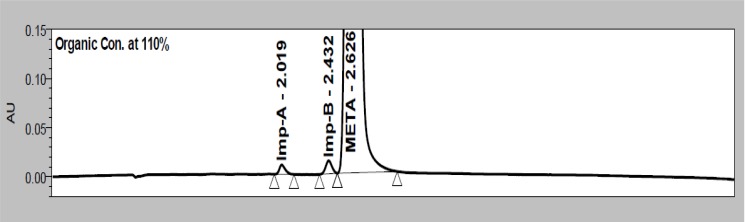
Chromatogram of mobile phase composition study (+10% for acetonitrile)

To determine the robustness of the method, the experimental conditions were deliberately changed. The resolution of META and imp-B was evaluated. The effect of change in flow rate ± 0.05mL/min (0.25 and 0.35 mL/min), column oven temperature ± 5°C (40 and 50°C), mobile phase pH ± 0.2 units (6.1 and 6.5 pH) and mobile phase composition ± 10% (for acetonitrile) were studied. During study other chromatographic conditions were kept the same as per the experimental section. In all the deliberately varied chromatographic conditions, all of the analytes were adequately resolved, and the order of elution remained unchanged. Robustness study obtained results are presented in Table 8. Figure 6 indicates that USP resolution 1.56 is also adequate for the peak separation in developed method.

### Stability of solution

Drug stability in pharmaceutical formulations is a function of storage conditions and chemical properties of the drug and its impurities. Conditions used in stability experiments should reflect situations likely to be encountered during actual sample handling and analysis. Stability data is required to show that the concentration and purity of the analyte in the sample at the time of analysis corresponds to the concentration and purity of the analyte at the time of sampling. META (1000 μg/mL) spiked solution (with 1 μg/mL of each impurity) was prepared in the diluent by leaving the test solutions at room temperature. The spiked solution was re-analyzed at 12h and 24h time intervals, assay and related substances were determinate for the compounds and compared against fresh sample. The sample solution did not show any appreciable change in assay and related substances value when stored at ambient temperature up to 24h, data are presented in Table 9. The results from solution stability experiments confirmed that sample solution was stable for up to 24h during assay and related substances determination.

### Filter compatibility

Filter compatibility was performed for PVDF 0.22 μm syringe filter (Millipore) and nylon 0.22 μm syringe filter (Pall Life sciences). To confirm the filter compatibility in proposed analytical method, filtration recovery experiment was carried out by sample filtration technique. Sample was filtered through both syringe filters. Assay and related substances were determined (in μg/mL) and compared against centrifuged sample. The sample solution was not showing any significant changes in assay and related substances with respect to centrifuged sample. Filter compatibility results are presented in Table 10, which indicates that both syringe filters are compatible with sample solution.

## Experimental

### Materials and Reagents

Metaxalone (99.9% w/w) working standard, Impurity-A (99.8% w/w) reference standard, Impurity-B (99.7% w/w) reference standard, placebo and Metaxalone tablets (Batch No.F027) were provided by Dr. Reddy’s laboratories Ltd., Hyderabad, India. HPLC grade acetonitrile and methanol were obtained from J.T.Baker (NJ., USA). GR grade orthophosphoric acid and triethylamine were obtained from Merck Ltd. (Mumbai, India). 0.22 μm nylon membrane filter and nylon syringe filters were purchased from Pall life science limited (India). 0.22 μm PVDF syringe filters were purchased from Millipore (India). High purity water was generated using Milli-Q Plus water purification system (Millipore^®^, Milford, MA, USA). All other chemicals used were of analytical grade.

### Equipment

Cintex digital water bath (Mumbai, India) was used for specificity study. Photo stability studies were carried out in a photo-stability chamber (SUNTEST XLS+, ATLAS, Germany). Thermal stability studies were performed in a dry air oven (Cintex, Mumbai, India).

### Chromatographic conditions

Analysis was performed on an Acquity UPLC^™^ system (Waters, Milford, USA), consisting of a binary solvent manager, sample manager and PDA (photo diode array) detector. System control, data collection and data processing were accomplished using Waters Empower^™^-2 chromatography data software. The chromatographic condition was optimized using an Acquity^®^ HSS-T3 (100 mm × 2.1 mm, 1.8 μm) column. The mobile phase consisted of a mixture of water, acetonitrile, methanol and triethylamine in the ratio of 50:25:25:0.1 (v/v/v/v), pH was adjusted to 6.3 with orthophosphoric acid and filtered through 0.22 μm nylon membrane filter. Mobile phase was used as a diluent. The final selected and optimized conditions were as follows: injection volume 1 μL, isocratic elution, flow rate 0.3 mL/min, column oven temperature at 45°C, detection wavelength 230 nm for assay and 205 nm for related substances determination. Under these conditions, the backpressure in the system was about 6,000 psi.

### System suitability solution preparation

Suitability solution was prepared by dissolving standard substances in diluent to obtain solution containing 1000 μg/mL of Metaxalone, 1 μg/mL of Imp-A and 1 μg/mL of Imp-B.

### Standard solution preparation

Standard solution was prepared by dissolving META working standard in diluent to obtain solution containing 1000 μg/mL for assay and 1μg/mL for related substances.

### Sample solution preparation

Sample solution was prepared by dissolving sample (20 tablets were crushed to fine powder by mortar and pestle) in diluent to obtain a solution containing 1000 μg/mL of Metaxalone (for assay and related substances). It was then filtered through 0.22 μm PVDF syringe filter and the filtrate was collected after discarding the first few milliliters.

## Conclusion

The rapid isocratic RP-UPLC method was developed for quantitative and related substances analysis of Metaxalone in pharmaceutical formulation. Satisfactory results were obtained from validation of the method. The run time (6 min) enabled rapid determination of META. This method exhibited an excellent performance in terms of sensitivity and speed. This stability-indicating method can be applied for the routine analysis of production samples and to check the stability of Metaxalone in bulk drug and formulation. Moreover, it can be applied for determination of assay, blend uniformity, content uniformity and in vitro dissolutions of pharmaceutical products, where sample load is higher and high throughput is essential for faster delivery of results.

## Figures and Tables

**Tab. 1.** Summary of mobile phase optimization

**MP-A**	**MP-B**	**Observation**
Water	ACN	Co-eluting peak of Imp-B and META.
0.1% aqueous TEA	ACN	Co-eluting peak of Imp-B and META.
0.1% aqueous TEA	ACN:MeOH (50:50 v/v)	Poor resolution (Imp-B and META). USP tailing more than 2.0 for META peak.
Mixture of water, ACN, MeOH and TEA [50:25:25:0.1 v/v/v/v]	N.A.	Poor resolution (Imp-B and META). USP tailing more than 3.0 for META peak.
Mixture of water, ACN, MeOH and TEA [50:25:25:0.1 v/v/v/v], pH adjusted 6.3 with OPA	N.A.	Poor resolution (Imp-B and META) and broad peak shape of META.

USP…United States Pharmacopoeia; MeOH…Methanol; TEA…Triethylamine; OPA…Orthophosphoric acid

**Tab. 2.** Summary of forced degradation results

**Degradation condition**	**Mass balance^#^**	**Purity**		**Observation**
		**Angle**	**Threshold**	
Control sample	99.9	0.055	0.261	NA
Acidic hydrolysis	99.2	0.095	0.285	No degradation observed
Alkaline hydrolysis	98.8	0.131	0.333	Significant degradation observed
Oxidation	98.5	0.075	0.284	No significant degradation observed
Water hydrolysis	99.5	0.055	0.258	No degradation observed
Thermal (solid)	100.1	0.055	0.261	No degradation observed
Exposed to UV at 254nm	100.3	0.055	0.259	No degradation observed

NA…Not applicable; **#**…% assay + % known impurities + area % unknown impurities

**Tab. 3.** System suitability results (system precision, method precision and intermediate precision)

**Test**	**Parameters**	**Imp-A (1μg/mL)**	**Imp-B (1μg/mL)**	**META (1μg/mL)**	**META (1000μg/mL)**
System Precision	Area % RSD	0.8	0.9	0.9	0.3

Precision (n=6)	USP resolution	NA	N.A.	2.34	NA
	USP tailing	NA	N.A.	1.12	1.19
	USP plate count	NA	N.A.	11975	11994

Intermediate precision (n=6)	USP resolution	NA	N.A.	2.32	NA
	USP tailing	NA	N.A.	1.13	1.15
	USP plate count	NA	N.A.	11848	12162

NA…Not applicable.

**Tab. 4.** Precision (n=6) and Intermediate precision (n=6) results

**Substance**	**Precision**		**Intermediate precision**	

	**Mean %**	**% RSD**	**Mean %**	**% RSD**
META (1000μg/mL)	99.3	0.9	99.5	1.1
Imp-A (1μg/mL)	0.098	2.4	0.101	2.3
Imp-B (1μg/mL)	0.103	2.7	0.097	2.6

**Tab. 5.** Accuracy results

**Substance**	**At 50% (n=3)**		**At 100% (n=3)**		**At 150% (n=3)**	

	**%Recovery**	**%RSD**	**%Recovery**	**%RSD**	**%Recovery**	**%RSD**

META (1000μg/mL)	100.7	0.9	99.5	1.2	99.1	1.3
META (1μg/mL)	94.7	3.1	97.9	3.7	98.9	3.4
Imp-A (1μg/mL)	95.2	4.8	103.1	3.6	99.4	4.1
Imp-B (1μg/mL)	102.6	3.6	104.4	4.3	97.6	3.9

**Tab. 6.** Regression statistics

**Compound**	**Linearity range (μg/mL)**	**Correlation coefficient (r^2^)**	**Linearity (Equation)**	**Y-intercept bias at 100%**
META	500 to 1500	0.9998	y = 3423.3(x) + 50372	1.451
META	0.1 to 2.0	0.9998	y = 28098(x) + 102.38	0.368
Imp-A	0.1 to 2.0	0.9998	y = 33041(x) − 208.53	−0.634
Imp-B	0.1 to 2.0	0.9994	y = 42025(x) + 813.06	1.887

**Tab. 7.** Results of LOD, LOQ and LOQ precision (n=6)

	**META**	**Imp-A**	**Imp-B**
LOD (μg/mL)	0.03	0.04	0.04
LOQ (μg/mL)	0.1	0.1	0.1
LOQ precision (% RSD)	4.5	4.9	5.7

**Tab. 8.** Robustness study results

**Condition**	**Parameters**	**Imp-A**	**Imp-B**	**META**
Normal methodology	USP resolution	−	−	2.34
	Retention time in min	2.350	2.827	3.159
	Assay % w/w	0.098	0.103	99.3

At flow rate 0.25 mL/min	USP resolution	−	−	2.31
	Retention time in min	2.814	3.392	3.790
	Assay % w/w	0.097	0.102	99.1

At flow rate 0.35 mL/min	USP resolution	−	−	2.51
	Retention time in min	2.015	2.418	2.716
	Assay % w/w	0.098	0.102	99.4

At 40°C column oven temp.	USP resolution	−	−	2.77
	Retention time in min	2.461	2.996	3.383
	Assay % w/w	0.099	0.104	99.7

At 50°C column oven temp.	USP resolution	−	−	2.07
	Retention time in min	2.243	2.669	2.954
	Assay % w/w	0.098	0.103	98.9

At mobile phase pH 6.1	USP resolution	−	−	1.70
	Retention time in min	2.034	2.449	2.660
	Assay % w/w	0.097	0.104	99.6

At mobile phase pH 6.5	USP resolution	−	−	2.36
	Retention time in min	2.375	2.853	3.201
	Assay % w/w	0.098	0.102	99.0

Mobile phase [-10% ACN]	USP resolution	−	−	2.77
	Retention time in min	2.792	3.316	3.876
	Assay % w/w	0.098	0.103	99.8

Mobile phase [+10% ACN]	USP resolution	−	−	1.56
	Retention time in min	2.019	2.432	2.626
	Assay % w/w	0.099	0.102	99.8

**Tab. 9.** Solution stability results

**Compound**	**0h**	**12h**	**24h**
META (1000μg/mL)	1005	1004	1003
Imp-A (1μg/mL)	1.003	1.007	1.008
Imp-B (1μg/mL)	0.975	0.967	0.978

**Tab. 10.** Filter compatibility results

**Compound**	**Centrifuged**	**PVDF filter 0.22μm**	**Nylon filter 0.22μm**
META (1000μg/mL)	1005	1004	1003
Imp-A (1μg/mL)	0.951	0.961	0.964
Imp-B (1μg/mL)	0.985	0.972	0.981
